# From the Wuhan-Hu-1 strain to the XD and XE variants: is targeting the SARS-CoV-2 spike protein still a pharmaceutically relevant option against COVID-19?

**DOI:** 10.1080/14756366.2022.2081847

**Published:** 2022-06-13

**Authors:** Matteo Pavan, Davide Bassani, Mattia Sturlese, Stefano Moro

**Affiliations:** Molecular Modeling Section (MMS), Department of Pharmaceutical and Pharmacological Sciences, University of Padova, Padova, Italy

**Keywords:** SARS-CoV-2, COVID-19, XE variant, XD variant, Spike protein, Mpro, Paxlovid

## Abstract

Since the outbreak of the COVID-19 pandemic in December 2019, the SARS-CoV-2 genome has undergone several mutations. The emergence of such variants has resulted in multiple pandemic waves, contributing to sustaining to date the number of infections, hospitalisations, and deaths despite the swift development of vaccines, since most of these mutations are concentrated on the Spike protein, a viral surface glycoprotein that is the main target for most vaccines. A milestone in the fight against the COVID-19 pandemic has been represented by the development of Paxlovid, the first orally available drug against COVID-19, which acts on the Main Protease (Mpro). In this article, we analyse the structural features of both the Spike protein and the Mpro of the recently reported SARS-CoV-2 variant XE, as well the closely related XD and XF ones, discussing their impact on the efficacy of existing treatments against COVID-19 and on the development of future ones.

## 1. Introduction

More than two years have now passed since the beginning of the COVID-19 pandemic, back in December 2019^1^^,^^2^. Caused by a beta coronavirus known as SARS-CoV-2 and characterised by para-influenzal symptoms such as fever, cough, and dyspnoea, this worldwide-spread disease has resulted in the death of more than 6 million people around the world, becoming one of the deadliest illnesses in human history^3^^,^^4^.

The SARS-CoV-2 virus was first identified in the Chinese city of Wuhan, where the pandemic was firstly spotted^5^. The genomic sequence of this virus (named Wuhan-Hu-1 from now on in the article) is80% identical to the one of the SARS-CoV virus^6^^,^^7^, which was responsible for the Severe Acute Respiratory Syndrome (SARS) that stroke the South East of Asia in 2002/2003, causing the death of about 800 patients over 9000 cases (10% death rate)^8^^,^^9^. The exact origin of the SARS-CoV-2 virus is still to this date unknown, however several pieces of evidence point out bat coronaviruses as closely related ancestors and the pangolin as the intermediate host before the human spillover^10–13^.

Soon after the original virus started spreading all over the world, several viral variants began to emerge^14^^,^^15^, especially in the poorest countries where public health measures such as social distancing and wearing surgical masks in public places were difficult to implement^16–18^. Most of the genome mutations that characterised these variants were concentrated in the S gene^19^, which encodes for the Spike protein, a surface glycoprotein that mediates the virus entry within the human cell through an interaction with the human ACE2 receptor^20^. Some of these mutations gathered the attention of the scientific community due to the selective advantage that they provided to the correspondent viral variants, regarding both the virus’ ability to infect human cells and to escape the immune system response^21^, gaining for these reasons the status of “variant of concern” (VOC).

The first SARS-CoV-2 variant to be labelled as VOC was the so-called Alpha variant (B.1.1.7). First identified in November 2020 in the Kent region of the United Kingdom and for this reason also known as the “English variant”, B.1.1.7 was estimated to be 29% more transmissible than the original virus^22^^,^^23^. Despite being more transmissible than other circulating viral strains^24^^,^^25^, and despite showing the first signs of reduced protection provided by vaccines, monoclonal antibodies, and convalescent sera^26–28^, the indication from clinical studies showed that the vaccine coverage (especially in those who had already completed the vaccination cycle) was still able to contain the impact of this variant on the sanitary system^29^.

Soon after the identification of the Alpha variant, a second VOC arose: the Delta variant (B.1.617.2), also known as the “Indian variant” due to being first detected in India in late 2020, rapidly overcame the Alpha variant becoming the dominant strain in the world, thanks to being 97% more transmissible than the original Wuhan virus^23^. The replacement of the less threatening Alpha variant with the Delta marked a significant change of pace in the pandemic trend, signing an increased burden for the health system^30^ and posing for the first time a serious threat to the protection provided by vaccines, convalescent sera, and monoclonal antibodies^31–33^ due to its increased ability to evade the immune system response^34^.

From November 2021 onwards, the Delta variant has been flanked by another VOC firstly identified in South Africa and defined as the Omicron variant (B.1.1.529)^35^. The rise of the Omicron variant, fuelled by a contemporary increase in transmissibility^36^ and immune evasion^37^, resulted in an unprecedented diffusion of the SARS-CoV-2 virus all over the world, being able to overcome even the protection provided by the full primary vaccination cycle and by most neutralising antibodies used in therapy^38–40^, thus inducing the introduction of a “booster dose” to bring the protective effect of vaccines back to adequate levels^41^^,^^42^.

In the face of this increasingly troublesome variant landscape, characterised by a progressive reduction of the efficacy of existing therapeutic options against COVID-19, a light at the end of the tunnel is possibly represented by the development and release on the market of Paxlovid. This therapeutic combination between the active principle Nirmatrelvir (also known as PF-07321332) and the pharmacokinetic enhancer Ritonavir, represents the first orally available drug specifically designed against SARS-CoV-2 virus^43^. Instead of targeting the Spike protein, this peptidomimetic entity is designed to inhibit the SARS-CoV-2 Main Protease (M^pro^) by covalently binding to Cysteine 145, one of the two components of the protease’s catalytic diad^44^. Clinical studies showed a remarkable therapeutic efficacy of this novel treatment, with Paxlovid being able to lower by 89% the risk of severe complications associated with COVID-19 infection in symptomatic, non-vaccinated, and non-hospitalized adult patients^45^.

Recently, three novel recombinant SARS-CoV-2 variants were identified in the United Kingdom: Xd, Xe, and Xf^46^. These variants are derived from the combination of the genomes of other major circulating variants, namely Delta, Omicron, and Omicron 2^46^^,^^47^. Among these three novel viral strains, particular worry is related to the XE variant, which derives from the recombination between two VOCs, Omicron and Omicron 2, and is supposed to be 13–20% more transmissible than the Omicron 2 variant^46^.

The rise of novel SARS-CoV-2 variants derived from the recombination of other threatening and heavily diffused ones poses a serious challenge in the fight against the COVID-19 pandemic since it could contribute to rendering existing therapeutic options inefficient or practically useless. To evaluate the impact that these recently reported recombinant variants could have on the efficacy of existing vaccines and treatments (Paxlovid, in particular), we performed a computational analysis to shed light on the key structural features that characterise both the Spike glycoprotein and the Main Protease of these novel viral strains. Moreover, we analysed the structural evolution of these two viral proteins throughout the pandemic, discussing the impact that mutations found on these strains had and will have on the efficacy of existing therapeutic options against COVID-19 and the development of future ones.

## 2. Materials and methods

The genome sequence for the SARS-CoV-2 virus and its variants, namely Delta, Omicron, XD, XE, and XF, was obtained through GenBank^48^. Accession codes for each of the considered genomes are reported in Table 1. In the case of newly discovered variants XD, XE, and XF, the sequence was chosen according to the one reported by the Nextclade project^48^.

**Table 1.** The genome sequences used in this work and their origin.

Organism	Isolate	Accession Code
SARS-CoV-2	“Wuhan-Hu-1”	NC_045512.2
SARS-CoV-2 “Delta”	“SARS-CoV-2/human/JPN/SARS-CoV-2”	OK091006.1
SARS-CoV-2 “Omicron”	“SARS-CoV-2/human/NLD/EMC-Omicron-1/2021”	OM287553.1
SARS-CoV-2 “XD”	“SARS-CoV-2/human/FRA/IHUCOVID-64762/2022”	OM990851.1
SARS-CoV-2 “XE”	/	OW018845.1
SARS-CoV-2 “XF”	/	OV940149.1

All the basic molecular modelling operations have been executed with the Molecular Operating Environment (MOE) suite (version 2019.01)^49^.

For what concerns the Spike protein, the approach chosen depended on the variant considered. For the wild-type (WT) Spike, the three-dimensional structure was retrieved from the Protein Data Bank (PDB code: 6ZDH^50^, method: cryo-EM, resolution: 3.70 Å), as well as for the Delta (PDB code: 7W9E^51^, method: cryo-EM, resolution: 3.10 Å), and the Omicron (PDB code: 7WPD^52^, method: cryo-EM, 3.18 Å) variants. The cited structures were all subjected to the same preparation procedure for molecular modelling.

After being downloaded, the *Structure Preparation* tool implemented in MOE was applied to rebuild the missing loops in the structures, the proper protonation state was assigned to each amino acid with the MOE “*Protonate 3 D”* application, and finally, the added hydrogens were minimised under the AMBER10:EHT^53^ force field implemented in MOE. Since experimentally resolved structures for the XD, XE, and XF variants are not available in public databases, the models considered for our study were created starting from the WT SARS-CoV-2 Spike coming from the PDB code 6ZDH by manually mutating the residues, exploiting the MOE *“Protein builder”* tool, and subjecting each protein to the preparation procedure reported above. For the realisation of the video reported in the Supplementary Materials, the program VMD 1.9.2^54^ (Visual Molecular Dynamics) was used.

For what concerns M^pro^, the protein sequences corresponding to the main protease were extracted from the whole genome sequence and aligned to the reference sequence (Wuhan-Hu-1) making use of the appropriate tool of MOE 2019.01. Subsequently, homology models for each variant were created making use of the “*Homology Model*” tool of MOE 2019.01, using the structure deposited in the Protein Data Bank with accession code 6Y2E (Crystal structure of the free enzyme of the SARS-CoV-2 main protease) as a template for model generation.

## 3. Results and discussion

### 3.1. Structural analysis of spike glycoprotein mutations found in SARS-CoV-2 XD, XE, and XF variants and their impact on hACE2 binding

The SARS-CoV-2 Spike protein (S) consists of a large biological entity formed by 1273 amino acids organised in different functional domains. The main role of the Spike protein is to mediate the virus entry into the host cell, with the principal and better-characterised mechanism being the pathway involving the binding to the human ACE2 receptor (hACE2)^55^, a membrane-bound enzyme that is widely expressed in various districts of the human body (from the endothelial cells of the blood vessels to kidneys, liver, intestine, lungs^56^, and cells of bronchial and nasal epithelia^57^).

The S protein, which works in a trimeric organisation, is divided into two main subunits, S1 and S2. The second of these has very important roles in spike protein trimerization and in mediating the virion entry into the host cell once the molecular contacts have been established. It is formed by relevant subdomains such as the fusion peptide (FP, residues 943–982, crucial for viral fusion to the host cell membrane), the transmembrane domain (TM, composed of 24 amino acids and deputed both to the anchoring of S protein to the viral membrane and the maintenance of the Spike trimeric organisation), and the cytoplasmatic fusion domain (CT, mediating virus-cell fusion).

The S1 subunit, instead, contains both the N-terminal and the C-terminal domains (NTD and CTD, respectively), which are involved in the binding to host cell receptors. Specifically, the CTD contains the receptor-binding domain (RBD, aminoacids 319–541), the region deputed to the binding with hACE2. This function is more precisely carried out by a particular RDB subdomain, called receptor-binding motif (RBM), which is formed by two beta-sheets (β5 and β6) composed of those residues which are in close contact with hACE2 (from 438 to 506)^58^^,^^59^.

Looking at all the S proteins of the different SARS-CoV-2 relevant variants, the RBD contains the highest “single-point mutations/sequence length” ratio in all cases. Examining the different SARS-CoV-2 variants discovered up to date, the Spike protein is surely the viral entity that has mutated the most in the evolutionary process of the virus^60^. Its exposition on the viral surface and its crucial function in viral cell entry make this protein the eligible target for the host immune system^61^.

The SARS-CoV-2 S protein has experienced several mutations in the past two years^48^, as reported in Tables 1 and 2 for the variants considered in our study. As can be noticed, variants such as Delta (but also Alpha and Beta, not specifically treated in this article) showed few mutations in the overall viral genome, and Spike protein displayed never more than a tenth of single-point changes. The game-changing event was the advent of the Omicron variant, much different from its previous analogs, with 30-single nucleotide mutations involving the S protein only. Many of these, such as K417N, T478K, and P614G were inherited from the previous lineages (mainly Beta and Delta), but other mutations were completely new, such as G339D, G446S, or E484A.

**Table 2.** List of all the single-point mutations affecting the SARS-CoV-2 Spike protein for all the variants considered in our study (Delta, Omicron, XE, XD, and XF).

Delta variant	Omicron variant	XD variant	XE variant	XF variant
T19R		T19R	T19R	
		A27S	A27S	
	A67V			A67V
	T95I	T95I		T95I
	G142D	G142D	G142D	
				Y145D
R158G		R158G		
	L212I	L212I		L212I
			V213G	
	G339D	G339D	G339D	G339D
	S371L	S371L	S371L	S371L
	S373P	S373P	S373P	S373P
	S375F	S375F	S375F	S375F
			T376A	
			D405N	
			R408S	
	K417N	K417N	K417N	K417N
	N440K	N440K	N440K	N440K
	G446S	G446S		G446S
L452R				
	S477N	S477N	S477N	S477N
T478K	T478K	T478K	T478K	T478K
	E484A	E484A	E484A	E484A
	Q493R	Q493R	Q493R	Q493R
	G496S	G496S		G496S
	Q498R	Q498R	Q498R	Q498R
	N501Y	N501Y	N501Y	N501Y
	Y505H	Y505H	Y505H	Y505H
	T547K	T547K		T547K
D614G	D614G	D614G	D614G	D614G
	H655Y	H655Y	H655Y	H655Y
	N679K	N679K	N679K	N679K
P681R	P681H	P681H	P681H	P681H
	N764K	N764K	N764K	N764K
	D796Y	D796Y	D796Y	D796Y
	N856K	N856K		N856K
	Q954H	Q954H	Q954H	Q954H
	N969K	N969K	N969K	N969K
	L981F	L981F		L981F

The mutations have been aligned to give a better perspective of the ones which have been conserved through the evolutionary process. The mutations involving the RBD have been highlighted in green, while the ones involving the RBM are coloured in cyan.

Most of the mutations listed in Table 2 have been related to higher infectivity, mainly due to a consequent gain of affinity for hACE2 or improved shielding from the immune cells. As evidence of this, the most successful vaccination campaigns for COVID-19 always involved the forced recognition of Spike protein from the patients’ immune cells^62^.

Specifically, mutations such as S371L, K417N, and Q493R were related to a diminished binding to the anti-coronavirus monoclonal antibody Casivirimab, while mutations like N440K and G446S confer resistance towards the antibody Imdevimab^63^. The combination of Casivirimab and Imdevimab has been used to treat COVID-19 patients but has demonstrated to be ineffective against the Omicron variant^64^. Other mutations external to the RBD have been linked to different outcomes, such as increased viral replication (Δ69–70^65^ and D614G^66^) and higher viral resistance (G339D and N440K^67^). In other scenarios, mutations have been reported to influence tropism of the S1/S2 cleavage, as in the cases of N679K and P681H^68^.

The majority of the mutations highlighted up to date on the SARS-CoV-2 S protein impact the binding with hACE2, as in the cases of S477N, Q498R, and N501Y^69^. These last mutations, as can be seen in Table 2, have been conserved from all the variants following Omicron, assessing their importance for the viral evolutionary process.

**Figure 1.**
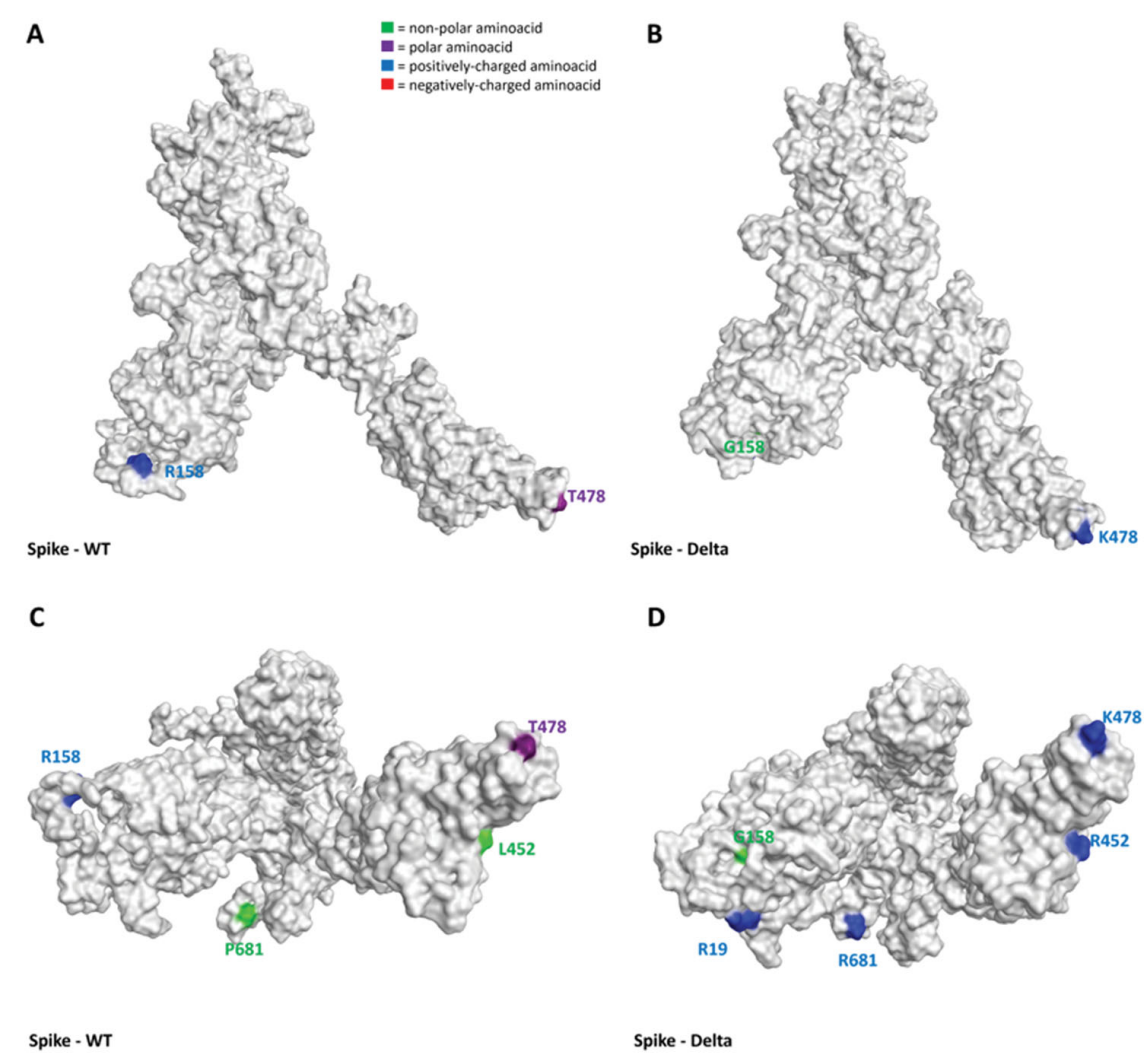
Representation of structural differences between the WT (taken from the Protein Data Bank^70^, PDB code: 6ZDH) and the Delta variant (retrieved from the PDB, code 7W9E^51^) of SARS-CoV-2 Spike protein. Panels A and B offer a front view of the comparison between the structures, while panels C and D shift the point of view to the bottom of the proteins. To give a clearer view of the mutations, only one monomer was considered to create the image. The amino acids involved in mutations are labelled in the figure and are coloured based on their kind, following the legend reported in panel A. Specifically, Gly, Ala, Val, Leu, Ile, Pro, Cys, Met, Phe, and Trp are considered non-polar amino acids (green), Asp and Glu represent the negatively-charged amino acids (red), and Lys, Arg, and His form the positively-charged amino acid group (blue). Finally, Ser, Thr, Asn, Gln, and Tyr are all considered polar amino acids (purple). All images were created and rendered using the Molecular Operating Environment (MOE) suite.

**Figure 2.**
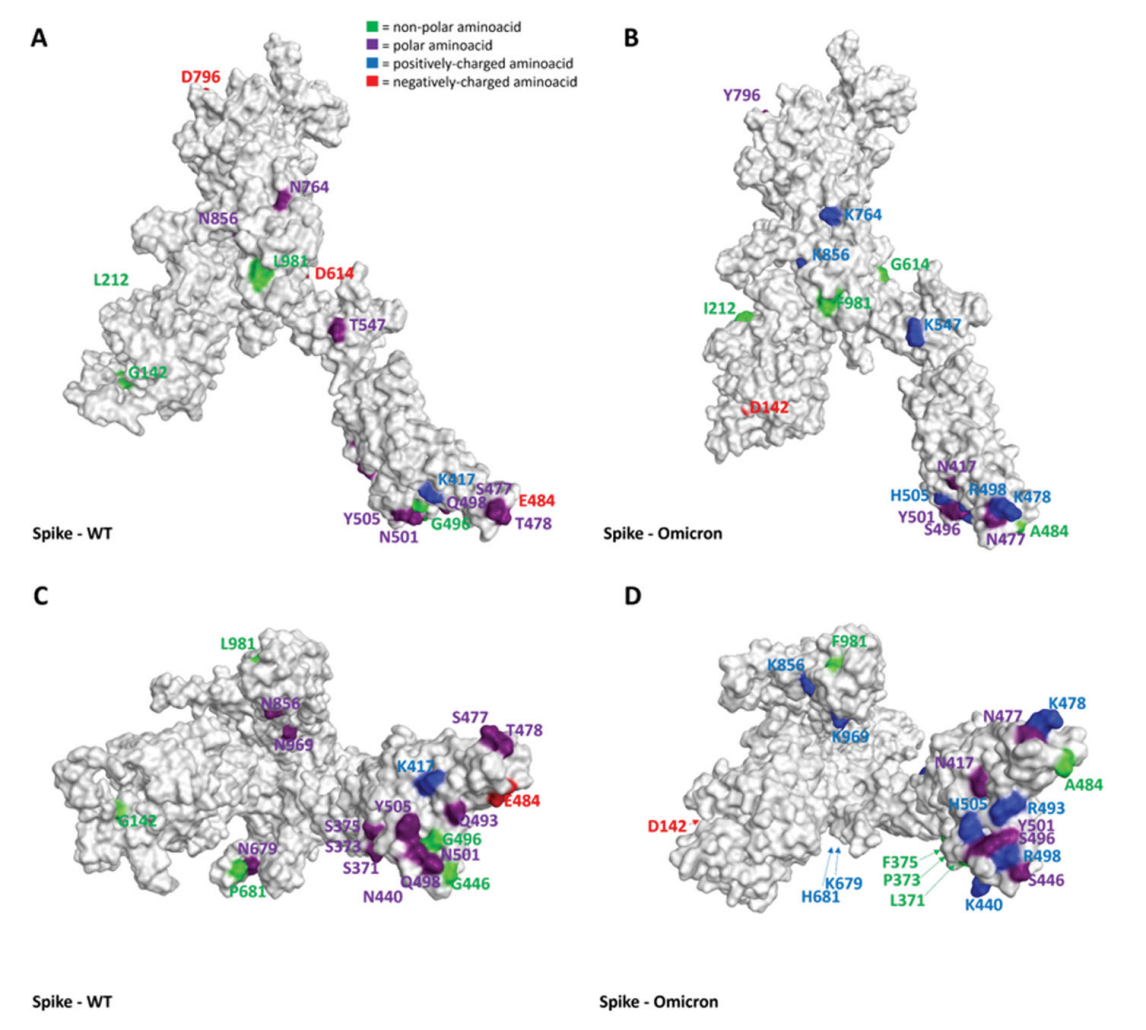
Representation of the differences between the WT (taken from PDB code: 6ZDH) and the Omicron variant (retrieved from the PDB, code 7WPD) of SARS-CoV-2 Spike protein. Panels A and B offer a front view of the comparison between the structures, while panels C and D shift the point of view to the bottom of the proteins. To give a clearer view of the mutations, only one monomer was considered to create the image. The amino acids involved in mutations are labelled in the figure and are coloured based on their kind, following the legend reported in panel A. Specifically, Gly, Ala, Val, Leu, Ile, Pro, Cys, Met, Phe, and Trp are considered non-polar amino acids (green), Asp and Glu represent the negatively-charged amino acids (red), and Lys, Arg, and His form the positively-charged amino acid group (blue). Finally, Ser, Thr, Asn, Gln, and Tyr are all considered polar amino acids (purple). All images were created and rendered using the Molecular Operating Environment (MOE) suite.

**Figure 3.**
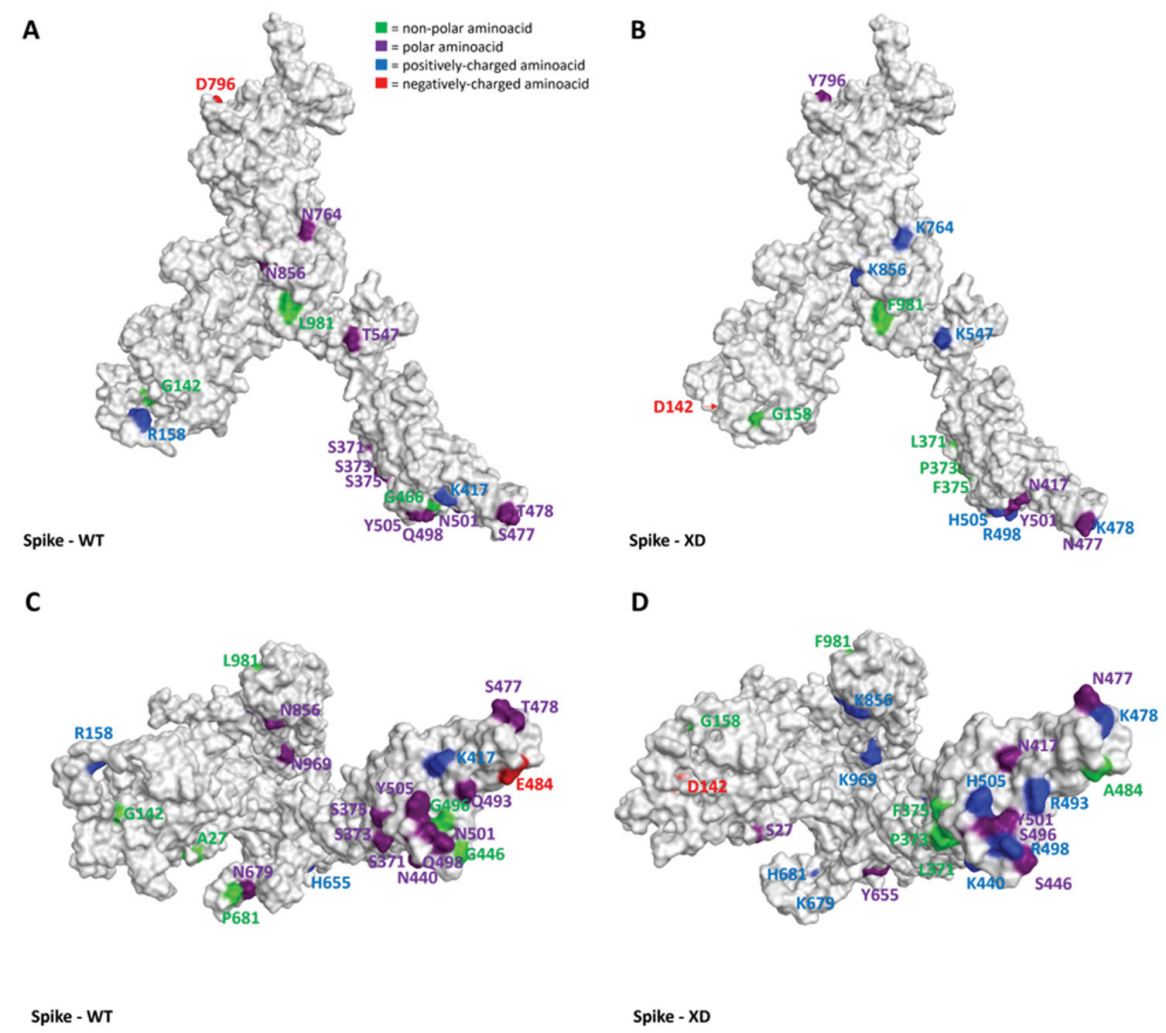
Representation of the differences between the WT (taken from PDB code: 6ZDH) and the XD a variant of SARS-CoV-2 Spike protein. Due to the lack of experimentally resolved structure of SARS-CoV-2 XD variant, the three-dimensional structure represented was obtained from the wild-type S protein coming from PDB code 6DZH, and then manually mutating the residues involved in the mutations (exploiting the MOE *“Protein builder”* tool). Panels A and B offer a front view of the comparison between the structures, while panels C and D shift the point of view to the bottom of the proteins. To give a clearer view of the mutations, only one monomer was considered to create the image. The amino acids involved in mutations are labelled in the figure and are coloured based on their kind, following the legend reported in panel A. Specifically, Gly, Ala, Val, Leu, Ile, Pro, Cys, Met, Phe, and Trp are considered non-polar amino acids (green), Asp and Glu represent the negatively-charged amino acids (red), and Lys, Arg, and His form the positively-charged amino acid group (blue). Finally, Ser, Thr, Asn, Gln, and Tyr are all considered polar amino acids (purple). All images were created and rendered using the Molecular Operating Environment (MOE) suite.

**Figure 4.**
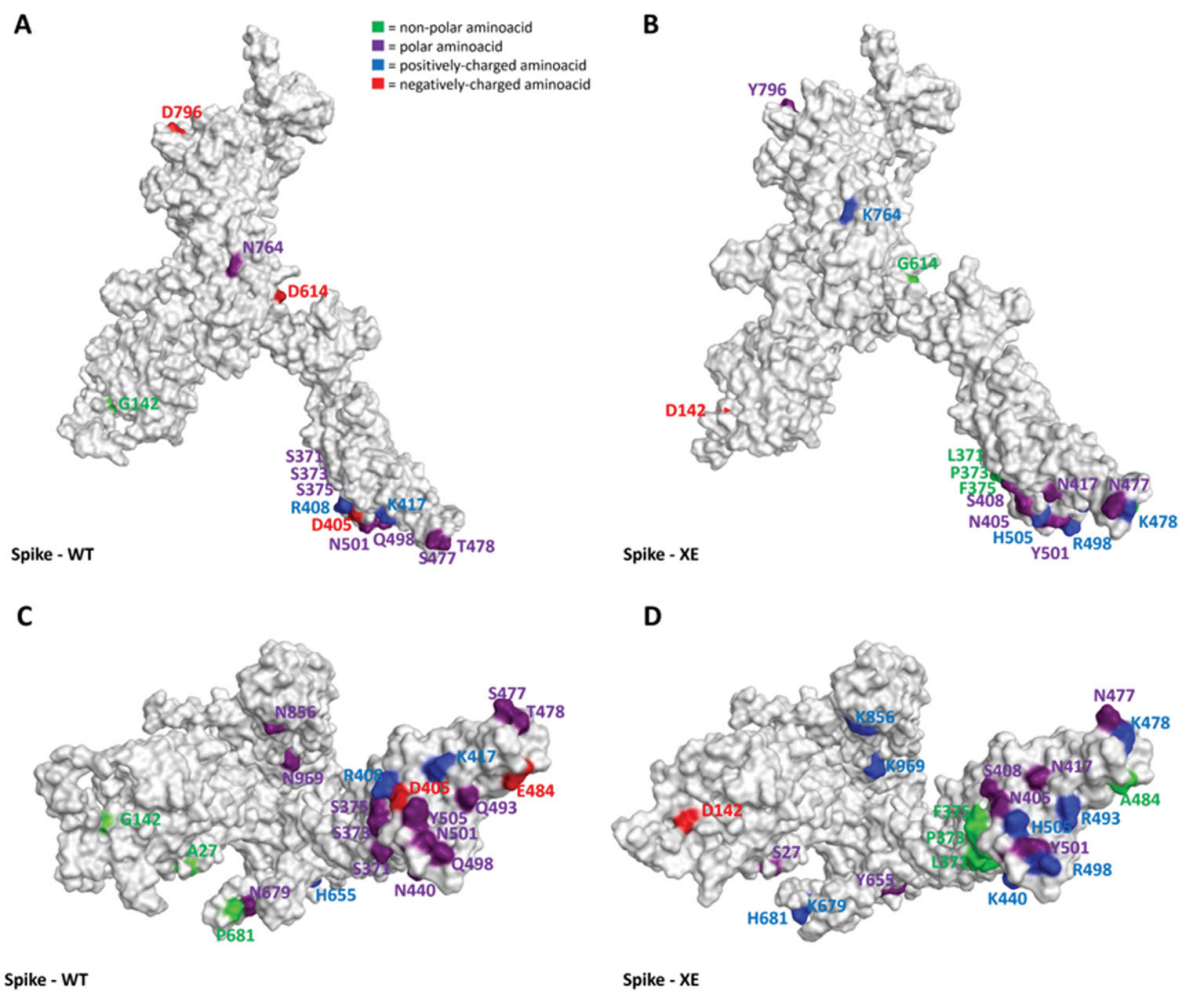
Representation of the differences between the WT (taken from PDB code: 6ZDH) and the XE variant of SARS-CoV-2 Spike protein. Due to the lack of experimentally resolved structure of the SARS-CoV-2 XE variant, the three-dimensional structure represented was obtained from the wild-type S protein coming from PDB code 6DZH, and then manually mutating the residues involved in the mutations (exploiting the MOE *“Protein builder”* tool). Panels A and B offer a front view of the comparison between the structures, while panels C and D shift the point of view to the bottom of the proteins. To give a clearer view of the mutations, only one monomer was considered to create the image. The amino acids involved in mutations are labelled in the figure and are coloured based on their kind, following the legend reported in panel A. Specifically, Gly, Ala, Val, Leu, Ile, Pro, Cys, Met, Phe, and Trp are considered non-polar amino acids (green), Asp and Glu represent the negatively-charged amino acids (red), and Lys, Arg, and His form the positively-charged amino acid group (blue). Finally, Ser, Thr, Asn, Gln, and Tyr are all considered polar amino acids (purple). All images were created and rendered using the Molecular Operating Environment (MOE) suite.

**Figure 5.**
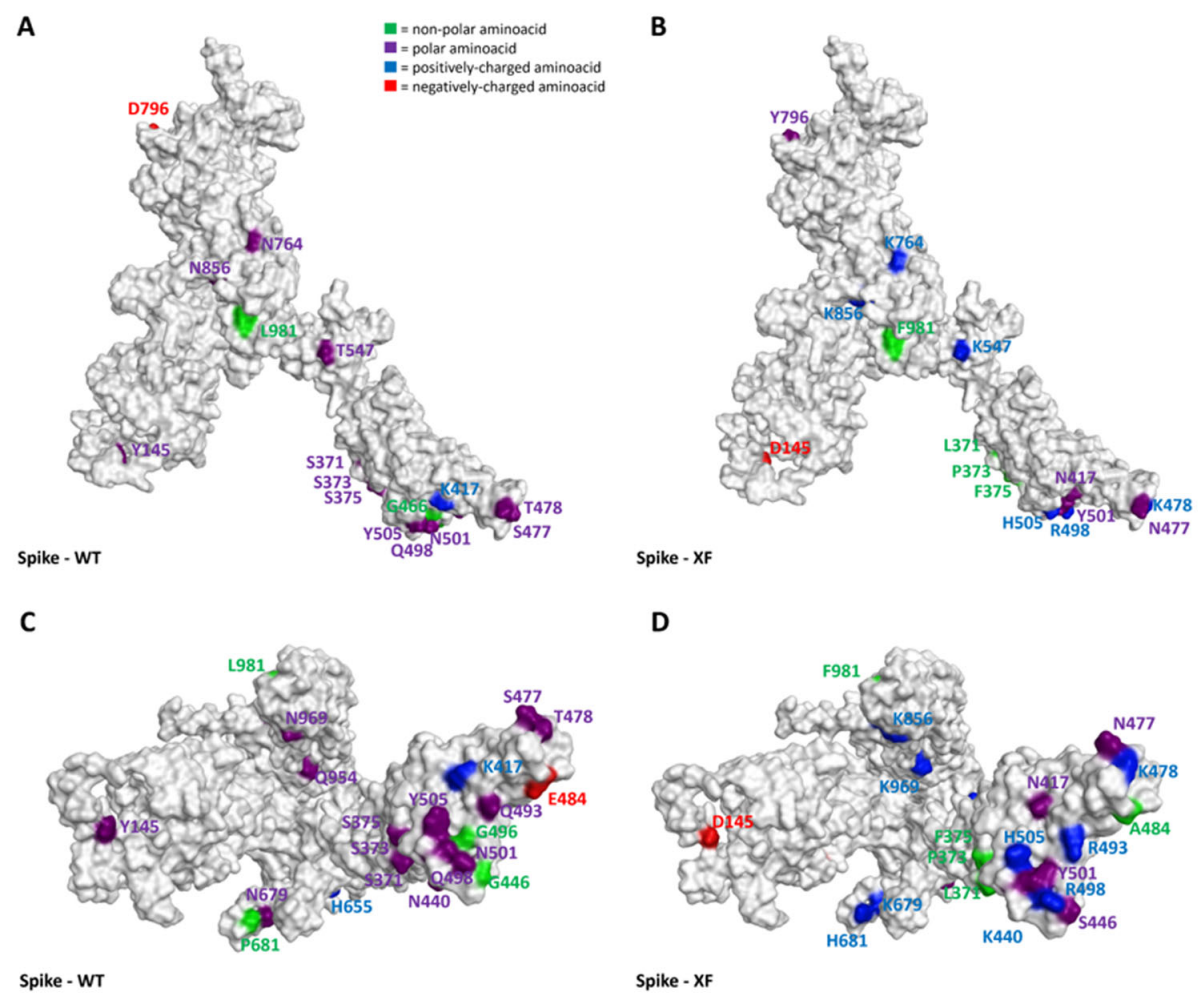
Representation of the differences between the WT (taken from PDB code: 6ZDH) and the XF variant of SARS-CoV-2 Spike protein. Due to the lack of experimentally resolved structure of the SARS-CoV-2 XF variant, the three-dimensional structure represented was obtained from the wild-type S protein coming from PDB code 6DZH, and then manually mutating the residues involved in the mutations (exploiting the MOE *“Protein builder”* tool). Panels A and B offer a front view of the comparison between the structures, while panels C and D shift the point of view to the bottom of the proteins. To give a clearer view of the mutations, only one monomer was considered to create the image. The amino acids involved in mutations are labelled in the figure and are coloured based on their kind, following the legend reported in panel A. Specifically, Gly, Ala, Val, Leu, Ile, Pro, Cys, Met, Phe, and Trp are considered non-polar amino acids (green), Asp and Glu represent the negatively-charged aminoacids (red), and Lys, Arg, and His form the positively-charged aminoacid group (blue). Finally, Ser, Thr, Asn, Gln, and Tyr are all considered polar amino acids (purple). All images were created and rendered using the Molecular Operating Environment (MOE) suite.

As can be seen in Figures 1–5 and video.mp4 (Supplementary Materials), the highest “number of mutations/sequence length” ratio is owned by the RDB, as previously mentioned. Indeed, taking Omicron as an example, among the 30 mutations in the overall 1273-residues structure, 15 are located just in the 222 residues-sequence forming the RBD. The insertions and the deletions (summarized in Table 3), on the other hand, are located far outside the hACE2-binding domain in all the variants examined, allowing us to assert that these mutations should not impact all the host cell recognition process.

**Table 3.** List of all the insertions and deletions operated by SARS-CoV-2 Spike protein for all the variants considered in our study (Delta, Omicron, XD, XE, and XF).

Delta variant	Omicron variant	XD variant	XE variant	XF variant
			Δ24-26	
	Δ69-70			Δ69-70
				Δ142-144
	Δ143-145			
Δ156-157		Δ156-157		
	Δ211	Δ211		Δ211
	ins214EPE	ins214EPE		ins214EPE

The mutations have been aligned to give a better perspective of the ones which have been conserved through the evolutionary process.

Interestingly, as depicted in the aforementioned figures, single-point mutations that are present in the RBD for all considered variants tend to progressively increase the positively-charged character of this protein region. Moreover, of all the changes operated by the evolutionary process of Spike protein, the very few mutations which transform a residue into a negatively-charged one (Asp or Glu) are always located away from the RBD (except for G339D, which is located in the posterior part of the RBD, away from the RBM that contacts hACE2). Indeed, in this region, the changes from polar amino acids to positively charged ones are abundant (N440K, T478K, Q498R, Y505H), and there are also cases in which non-polar residues transform into polar ones (e.g. G446S and G496S, which are conserved in all examined post-Omicron variants except for the XE one). Another conserved structural feature across all post-Omicron variants is also the fact that E484, located in the RBM, mutates into an alanine, while a peculiar mutation exclusive to the XE variant is represented by the transformation of D405 into an asparagine. Taken together, all these pieces of evidence converge in assessing that an increase in the polar characteristics of the RBD (more specifically, the RBM), with particular relevance to an increase in the number of positively charged amino acids, could be the mechanism adopted by SARS-CoV-2 to continuously increase its infectivity through an increase in the interaction with hACE2.

**Figure 6.**
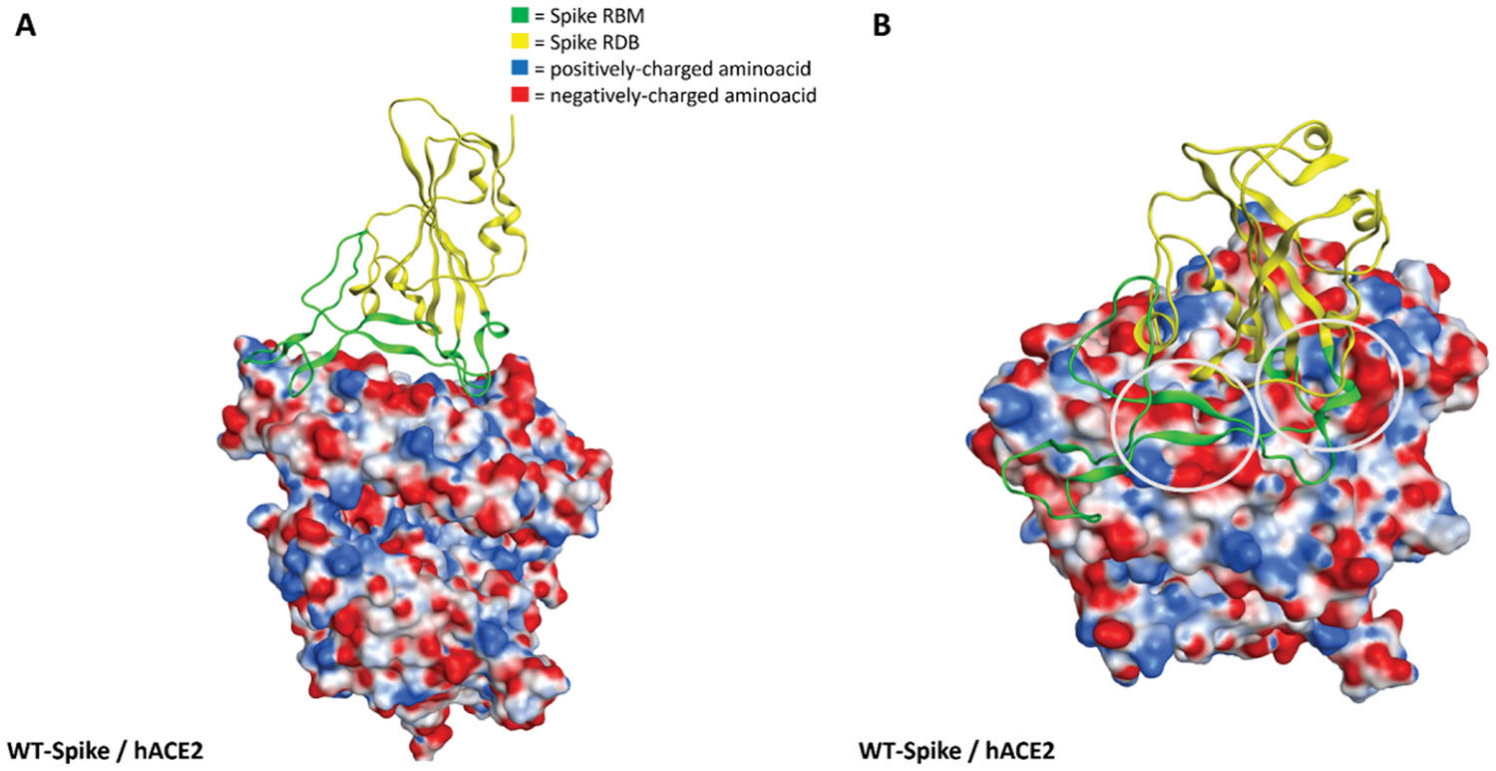
Representation of the interaction between WT-Spike receptor-binding domain (RBD) and hACE2 (coming from PDB code: 6M0J^58^). The Spike RBD is coloured in yellow, while the receptor-binding motif (RBM) is coloured in green. The hACE2 surface is coloured according to the electrostatic properties of underlying residues (blue, positively-charged regions, red, negatively-charged regions, white, neutral regions). Panel A offers a lateral view of the complex, while panel B focuses the attention on a top-lateral perspective. As can be seen from panel B, the hACE2 regions in contact with Spike-RBM are prevalently negatively-charged (red color): concerning this, for visualisation purposes, the most extended negative regions at the Spike-hACE2 interface have also been highlighted with grey circles.

To further support this evidence, in Figure 6 we reported the electrostatic surface of hACE2 complexed with WT-Spike RBD highlighting the prevalence of negative charge on the surface facing Spike RBM (coloured in green in the image). Coherently, the only mutation present in the RBD in which a positively-charged residue shift into a polar one (K417N) has been reported to reduce the affinity with hACE2^26^. It is worth noting that other hACE2-independent entry routes for SARS-CoV-2 have been described in literature^71^^,^^72^, but the lack of reliable structural information about the interaction with the target at the present date hampers and limits the possibility to analyse and discuss the impact that these mutations could have on them in a meaningful way. However, it cannot be excluded that this mutation pattern and other future Spike mutations could also impact these other entry pathways, contributing to making them more relevant for the ability of SARS-CoV-2 to infect human cells and increasing its overall infectivity.

### 3.2. Structural analysis of main protease mutations found in SARS-CoV-2 XD, XE, and XF variants and their impact on the recognition of known inhibitors

The main protease M^pro^, also known as 3 C-like protease or 3CL^pro^, is a cysteine peptidase that is essential for the replication cycle of SARS-CoV-2^73^^,^^74^. Its catalytic activity revolves around the processing of two overlapping polyproteins, namely pp1a and pp1ab, which leads to the formation of 16 mature non-structural proteins (NSPs)^75^. Composed of 306 amino acids, the SARS-CoV-2 M^pro^ shares 96% sequence identity and a highly conserved three-dimensional structure with the SARS-CoV M^pro^ (0.53 Å R.M.SD between PDB entries 6Y2E and 2BX4)^76^^,^^77^. Although a dynamic equilibrium between a monomeric and a dimeric form exists, only the dimer is responsible for the protease’s enzymatic activity^78^^,^^79^. Each protomer composing the catalytically active dimer is composed of three different domains: the chymotrypsin-like β-barrel domains I (residues 1–99) and II (residues 100–182), which comprehend the substrate-binding site and directly control the catalytic event, and the extra α-helical domain III (residues 198–306), which is connected to the remaining domains through a 16 residues loop and is involved in the dimerisation process, thus playing an indirect role in the regulation of M^pro^ catalytic activity^78^^,^^80^.

The catalytic site is a shallow, solvent-exposed cavity that is formed by several sub-pockets that are responsible for the recognition of various residues composing the substrate peptide sequences^76^^,^^80^. Concerning this, particularly important is the conserved sequence Gln↓-Ser, where Gln↓- indicates the glutamine residue that precedes the cleavage site^81^.

Despite its peculiar structural features, which make it a difficult target for drugs, rational structure-based approaches such as Molecular Docking^82^ and Molecular Dynamics^83^ have proven to be useful tools in the identification and characterisation of M^pro^ small molecule inhibitors, leading to the discovery of both covalent and non-covalent lead compounds^84^^,^^85^. Further reinforcing the importance of the Main Protease as a key drug target against COVID-19, is the discovery and approval by regulatory agencies of Nirmatrelvir, the first drug specifically designed against SARS-CoV-2 to enter the market^43^.

Due to its pivotal role in the virus replication cycle, the Main Protease is, on the contrary to Spike, particularly conserved in its primary sequence and its three-dimensional structural features among different viral strains. Taking a closer look at the main proteases from previously mentioned SARS-CoV-2 variants, only one out of 306 amino acids is mutated compared to the reference sequence, precisely residue 132, which is a proline in the case of Wuhan-Hu-1, Delta, and XD viral strains, while it is mutated to histidine in the case of Omicron, XE, and XF variants.

**Figure 7.**
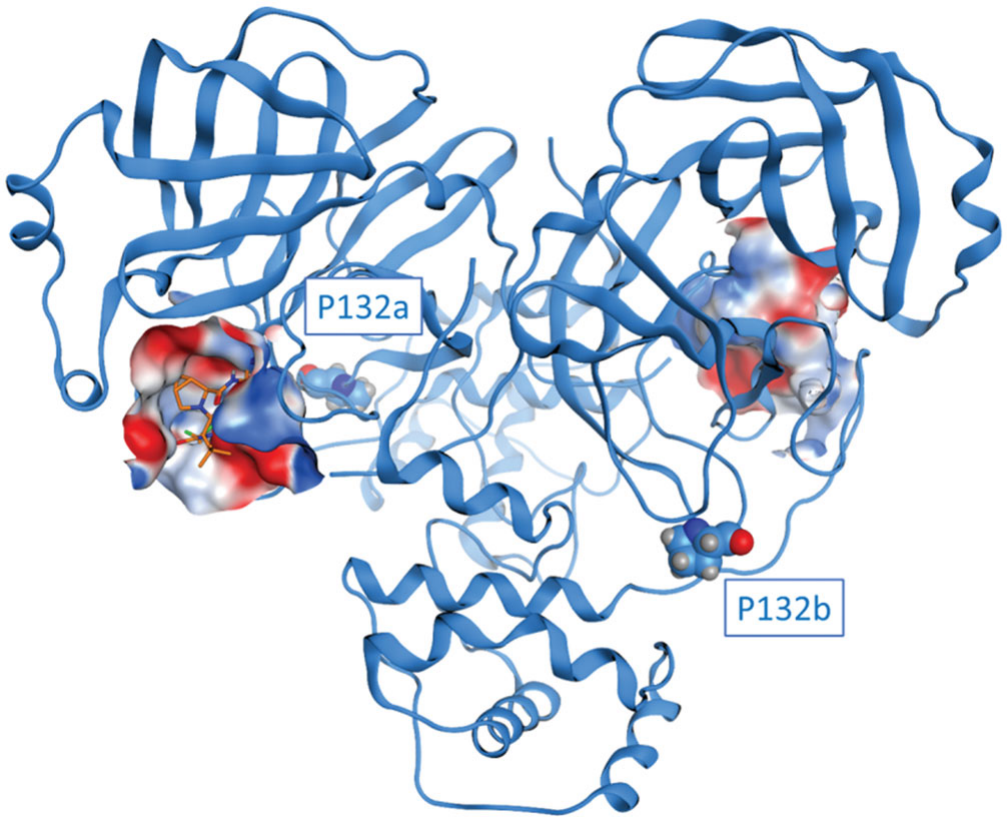
The structure of SARS-CoV-2 M^pro^ (PDB ID: 6Y2E) in its free form. The protein is depicted in blue ribbons, while the mutated residue P132 in comparison with considered SARS-CoV-2variants (Delta, Omicron, XD, XE, and XF) is highlighted and depicted as a CPK model. For visual reference, Nirmatrelvir (also known as PF-07321332, commercial name Paxlovid) from structure 7RFS is also shown in the picture, alongside the binding site surface coloured according to electrostatic properties.

As can be seen in Figure 7, this mutated residue is located outside the substrate-binding site, specifically in a turn that precedes the sequence leading to the oxyanion loop (residues 138–145), which is a vital part of the catalytic machinery that is responsible for the processing of substrate peptides^76^. Although the position of such mutation could suggest a possible destabilisation of the catalytic site related to an alteration of the enzymatic activity of the protease, a visual inspection of the surroundings of residue 132 suggests that this mutation should not affect in any way the stability of the three-dimensional structure of the protease, thereby not harming its ability to correctly process the substrate.

**Figure 8.**
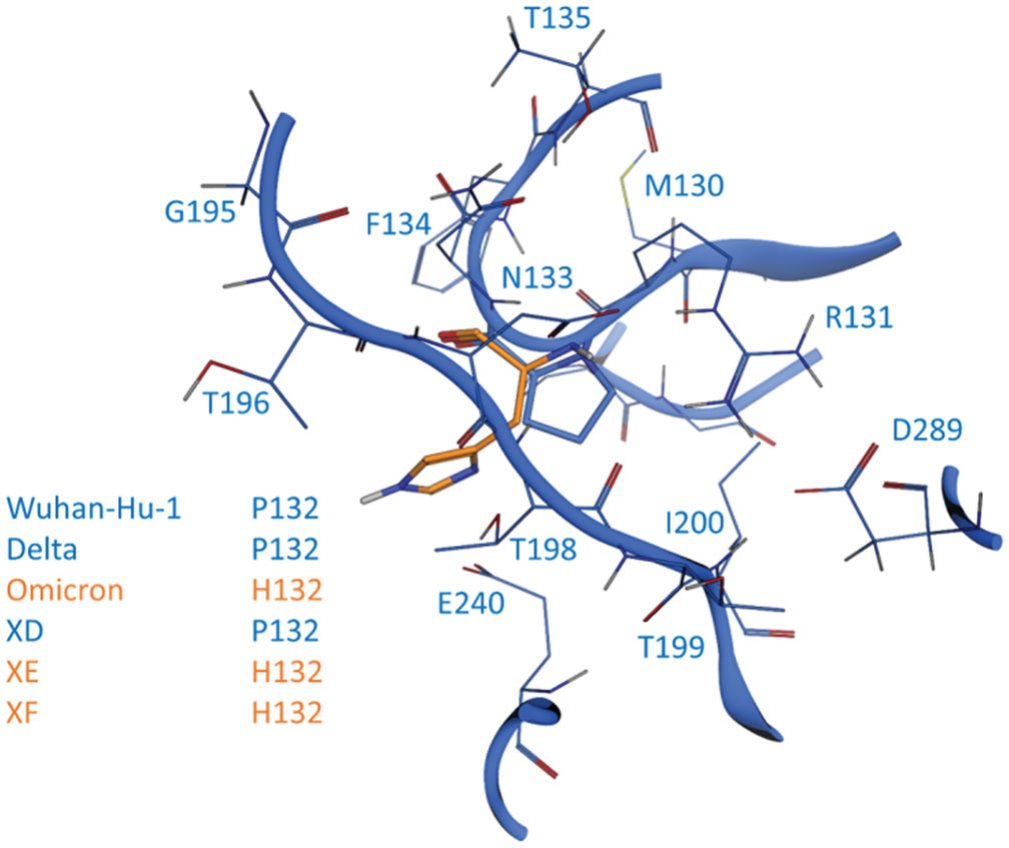
Comparison between SARS-CoV-2 3CL protease (M^pro^) from crystal structure 6Y2E (blue) and homology models of M^pro^ from five different SARS-CoV-2 variants, reported in Table 1: the focus is on residue 132 (either proline or a histidine) of SARS-CoV-2 M^pro^ and homology models of Delta, Omicron, XD, XE and XF variants M^pro^.

As can be seen in Figure 8, indeed, the proline residue is not involved in any intermolecular interaction relevant to the structural stability of the protease, suggesting that its only role could be limited to a joint between more relevant residues such as R131, which mediates several interactions through its sidechain guanidium group (specifically, a salt bridge with both D289 and D197, and a hydrogen bond with the backbone of T198) and its backbone (a hydrogen bond between its backbone amide proton and the amide carbonyl oxygen of T135 and another one between its carbonyl oxygen and the amide proton of F134), and N133, which is itself involved in a network of intermolecular interactions with both its backbone (hydrogen bond between its amide proton and the carboxyl oxygen of D197) and its sidechain (the amide proton donates to the carbonyl oxygen of G195 while the carbonyl oxygen receives from the hydroxyl group of T135). These structural insights are confirmed also by a functional screening performed by Flynn et al., which showed that mutations at this position, especially the P132H found in these viral variants, are generally well-tolerated, while mutations of both R131 and N133 drastically reduce or even abolish the catalytic activity of the protease^86^.

Concerning the relevance of this mutation for the efficacy of existing treatments and the development of future ones, a recent study from Greasley et al. reported the crystal structure of Nirmatrelvir in complex with the main protease from three different viral variants that presented a mutation on M^pro^^87^. The analysed mutations included the P132H, which characterises both the Omicron SARS-CoV-2 variant and the recently found XE and XF. Greasley and collaborators established that the P132H mutation does not affect the affinity of Nirmatrelvir for the main protease catalytic site, thereby indicating the same data would be extendable also to XE and XF variants considering that they share the same P132H mutation as the Omicron variant.

**Figure 9.**
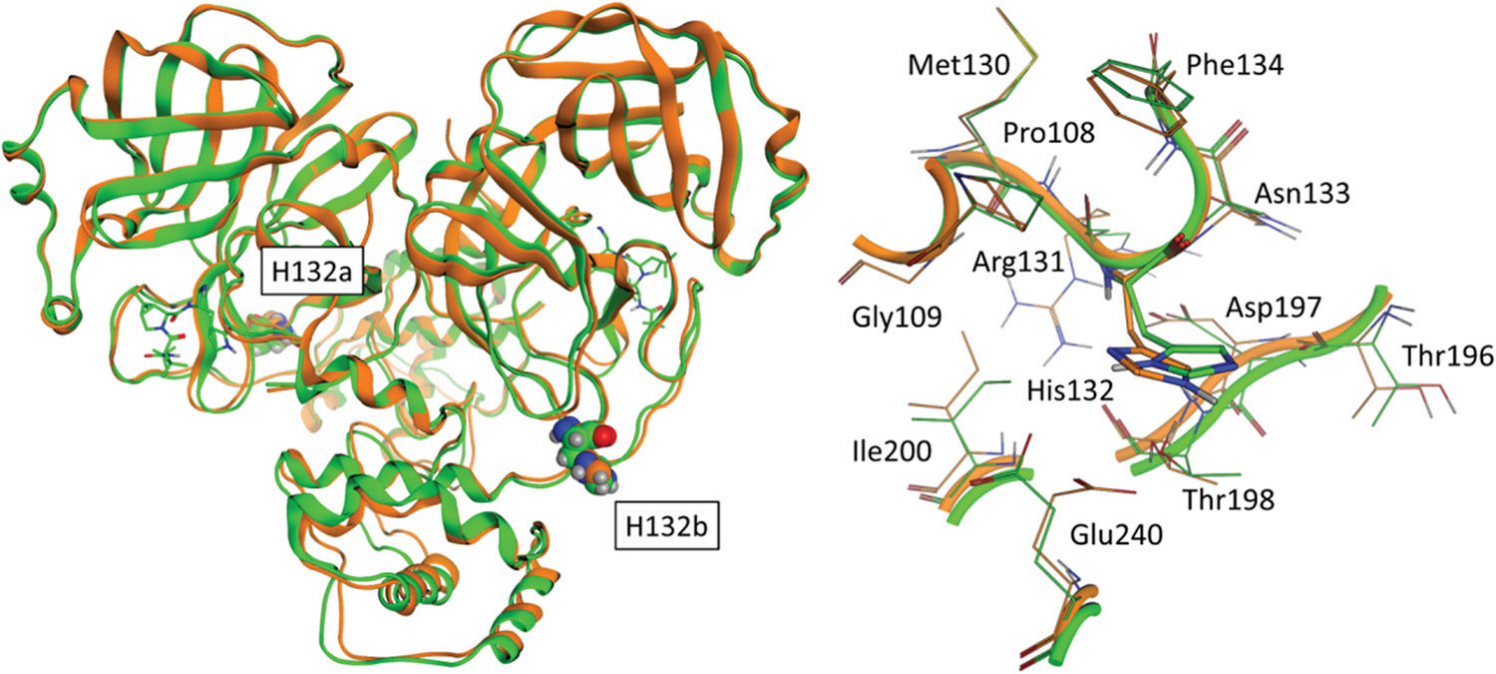
This panel reports the structural superposition between the crystal structure of SARS-CoV-2 Omicron variant M^pro^in complex with Nirmatrelvir (PDB ID: 7TLL, green) and the homology model of the XE/XF variant based on the unliganded state of the original SARS-CoV-2 virus (orange). In panel A, the whole protease structure is shown in the ribbon: for visual reference, both Nirmatrelvir and H132 are reported in liquorice and CPK models respectively. In panel B, a focussed view of residue 132 and its surrounding residues is reported.

As can be seen in Figure 9, although our homology model of the XE/XF variant is based on the structure 6Y2E, which represents the SARS-CoV-2 main protease in its free/unliganded form, there is an almost perfect structural superposition between our homology model and the experimentally resolved structure of the complex between the M^pro^ from the Omicron variant and Nirmatrelvir (PDB ID: 7TLL), as is also quantitatively assessed by the 0.67 Å R.M.SD between the two structures after optimal superimposition of the backbone. The congruence between our structural prediction and the experimental data supports the idea that the overall fold of the main protease is conserved across several variants and that the structural effect that residue mutations could have on the effectiveness of the main protease inhibitor could be accurately predicted through the combination of computational techniques such as homology modelling, molecular docking, and molecular dynamics. Moreover, based on available structural information, the high degree of structural similarity between the main proteases is not only shared by variants of the SARS-CoV-2 virus but also by other coronaviruses such as bat coronavirus^13^, the Porcine transmissible Gastroenteritis virus (TGEV)^78^, Human coronavirus strain 229E (HCoV)^80^, Infectious bronchitis virus (IBV)^88^and MERS-CoV^89^, thereby validating the pursue of novel M^pro^ inhibitors that could act as pan-coronaviral drugs and help to prevent future coronavirus associated pandemics.

## 4. Conclusions

The recent emergence of novel recombinant SARS-CoV-2 variants, namely XD, XE, and XF, poses a serious threat to the efficacy of existing therapeutic options against COVID-19. In the face of a continuous evolution of the SARS-CoV-2 genome under an evolutionary pressure opposed by the development of vaccines and by the natural immunity induced by infections, the more recent viral variants have increased both their infectiveness and their ability to escape the immune response. The structural analysis reported in this article depicts a scenario where the Spike protein, which is responsible for the ability of the virus to infect human cells by interaction with the hACE2 receptor, is the viral entity that is accumulating the highest number of mutations in an attempt to increase its affinity towards the hACE2 and decrease the one towards antibodies, while the main protease M^pro^, a key enzyme for the virus replication cycle, is still practically identical to the wild-type virus. The different behaviour of these two proteins in response to SARS-CoV-2 genome evolution could be vital not only for the development of efficient therapies against COVID-19 but, considering the striking structural similarities between the main protease from different viruses, also in the development of pan-coronaviral drugs that could prevent the development of future coronavirus-associated pandemics.

## Supplementary Material

Supplemental Material

## References

[1] Lu H, Stratton CW, Tang YW. Outbreak of pneumonia of unknown etiology in Wuhan, China: the mystery and the miracle. J Med Virol 2020;92:1704–2. 10.1002/jmv.25678 PMC7166628 31950516

[2] Heymann DL, Shindo N. COVID-19: what is next for public health? Lancet 2020;395:542–5. 32061313 10.1016/S0140-6736(20)30374-3 PMC7138015

[3] COVID Live. Coronavirus Statistics - Worldometer. https://www.worldometers.info/coronavirus/

[4] Guarner J. Three emerging coronaviruses in two decades: the story of SARS, MERS, and now COVID-19. Am J Clin Pathol 2020;153:420–1. 32053148 10.1093/ajcp/aqaa029 PMC7109697

[5] Zhou F, Yu T, Du R, et al. Clinical course and risk factors for mortality of adult inpatients with COVID-19 in Wuhan, China: a retrospective cohort study. Lancet 2020;395:1054–62. 32171076 10.1016/S0140-6736(20)30566-3 PMC7270627

[6] Bolcato G, Bissaro M, Pavan M, et al. Targeting the coronavirus SARS-CoV-2: computational insights into the mechanism of action of the protease inhibitors lopinavir, ritonavir and nelfinavir. Sci Rep 2020;10:20927. 33262359 10.1038/s41598-020-77700-z PMC7708625

[7] Gorbalenya AE, et al. The species Severe acute respiratory syndrome-related coronavirus: classifying 2019-nCoV and naming it SARS-CoV-2. Nat. Microbiol 2020;545:536–44. 10.1038/s41564-020-0695-z PMC7095448 32123347

[8] Ksiazek TG, Erdman D, Goldsmith CS, et al.; SARS Working Group. A novel coronavirus associated with severe acute respiratory syndrome. N Engl J Med 2003;348:1953–66. 12690092 10.1056/NEJMoa030781

[9] Peiris JSM, Chu CM, Cheng VCC, et al. Clinical progression and viral load in a community outbreak of coronavirus-associated SARS pneumonia: a prospective study. Lancet 2003;361:1767–72. 12781535 10.1016/S0140-6736(03)13412-5 PMC7112410

[10] Li W, Shi Z, Yu M, et al. Bats are natural reservoirs of SARS-like coronaviruses. Science 2005;310:676–9. 16195424 10.1126/science.1118391

[11] Andersen KG, Rambaut A, Lipkin WI, et al. The proximal origin of SARS-CoV-2. Nat Med 2020;26:450–2. *2020* 32284615 10.1038/s41591-020-0820-9 PMC7095063

[12] Temmam S, Vongphayloth K, Baquero E, et al. Bat coronaviruses related to SARS-CoV-2 and infectious for human cells. Nature 2022;604:330–6. 35172323 10.1038/s41586-022-04532-4

[13] Pavan M, Bassani D, Sturlese M, Moro S. Bat coronaviruses related to SARS-CoV-2: what about their 3CL proteases (MPro)? J Enzyme Inhib Med Chem 2022;37:1077–82. 35418253 10.1080/14756366.2022.2062336 PMC9037175

[14] Forster P, Forster L, Renfrew C, Forster M. Phylogenetic network analysis of SARS-CoV-2 genomes. Proc Natl Acad Sci U S A 2020;117:9241–3. 32269081 10.1073/pnas.2004999117 PMC7196762

[15] Harvey WT, Carabelli AM, Jackson B, COVID-19 Genomics UK (COG-UK) Consortium, et al. SARS-CoV-2 variants, spike mutations and immune escape. Nat Rev Microbiol 2021;19:409–24. 34075212 10.1038/s41579-021-00573-0 PMC8167834

[16] Chu DK, Akl EA, Duda S, COVID-19 Systematic Urgent Review Group Effort (SURGE) study authors, et al. Physical distancing, face masks, and eye protection to prevent person-to-person transmission of SARS-CoV-2 and COVID-19: a systematic review and meta-analysis. Lancet 2020;395:1973–2020. 32497510 10.1016/S0140-6736(20)31142-9 PMC7263814

[17] Hellewell J, Abbott S, Gimma A, et al. Feasibility of controlling COVID-19 outbreaks by isolation of cases and contacts. Lancet Glob. Heal 2020;8:e488–e496. 10.1016/S2214-109X(20)30074-7 PMC7097845 32119825

[18] Oran DP, Topol EJ. Prevalence of asymptomatic SARS-CoV-2 infection. Ann Inter Med 2020;173:362–8. 10.7326/M20-3012 PMC7281624 32491919

[19] Garcia-Beltran WF, Lam EC, St Denis K, et al. Multiple SARS-CoV-2 variants escape neutralization by vaccine-induced humoral immunity. Cell 2021;184:2372–83.e9. 33743213 10.1016/j.cell.2021.03.013 PMC7953441

[20] Hoffmann M, Kleine-Weber H, Schroeder S, et al. SARS-CoV-2 cell entry depends on ACE2 and TMPRSS2 and is blocked by a clinically proven protease inhibitor. Cell 2020;181:271–80.e8. 32142651 10.1016/j.cell.2020.02.052 PMC7102627

[21] Li Q, Wu J, Nie J, et al. The impact of mutations in SARS-CoV-2 spike on viral infectivity and antigenicity. Cell 2020;182:1284–94.e9. 32730807 10.1016/j.cell.2020.07.012 PMC7366990

[22] Frampton D, Rampling T, Cross A, et al. Genomic characteristics and clinical effect of the emergent SARS-CoV-2 B.1.1.7 lineage in London, UK: a whole-genome sequencing and hospital-based cohort study. Lancet Infect Dis 2021;21:1246–56. 33857406 10.1016/S1473-3099(21)00170-5 PMC8041359

[23] Campbell F, Archer B, Laurenson-Schafer H, et al. Increased transmissibility and global spread of SARSCoV- 2 variants of concern as at June 2021. Eurosurveillance 2021;26:1–6. 10.2807/1560-7917.ES.2021.26.24.2100509 PMC8212592 34142653

[24] Davies NG, Abbott S, Barnard RC, CMMID COVID-19 Working Group, et al. Estimated transmissibility and impact of SARS-CoV-2 lineage B.1.1.7 in England. Science 2021;372:eabg3055. 33658326 10.1126/science.abg3055 PMC8128288

[25] Volz E, Mishra S, Chand M, COVID-19 Genomics UK (COG-UK) consortium, et al. Assessing transmissibility of SARS-CoV-2 lineage B.1.1.7 in England. Nature 2021;593:266–9. 33767447 10.1038/s41586-021-03470-x

[26] Collier DA, De Marco A, Ferreira IATM, COVID-19 Genomics UK (COG-UK) Consortium, et al. Sensitivity of SARS-CoV-2 B.1.1.7 to mRNA vaccine-elicited antibodies. Nature 2021;593:136–41. 33706364 10.1038/s41586-021-03412-7 PMC7616976

[27] Chen RE, Zhang X, Case JB, et al. Resistance of SARS-CoV-2 variants to neutralization by monoclonal and serum-derived polyclonal antibodies. Nat Med 2021;27:717–26. 33664494 10.1038/s41591-021-01294-w PMC8058618

[28] Wang P, Nair MS, Liu L, et al. Antibody resistance of SARS-CoV-2 variants B.1.351 and B.1.1.7. Nature 2021;593:130–5. 33684923 10.1038/s41586-021-03398-2

[29] Haas EJ, Angulo FJ, McLaughlin JM, et al. Impact and effectiveness of mRNA BNT162b2 vaccine against SARS-CoV-2 infections and COVID-19 cases, hospitalisations, and deaths following a nationwide vaccination campaign in Israel: an observational study using national surveillance data. Lancet 2021;397:1819–29. 33964222 10.1016/S0140-6736(21)00947-8 PMC8099315

[30] Twohig KA, Nyberg T, Zaidi A, COVID-19 Genomics UK (COG-UK) consortium, et al. Hospital admission and emergency care attendance risk for SARS-CoV-2 delta (B.1.617.2) compared with alpha (B.1.1.7) variants of concern: a cohort study. Lancet Infect Dis 2022;22:35–42. 34461056 10.1016/S1473-3099(21)00475-8 PMC8397301

[31] Lopez Bernal J, Andrews N, Gower C, et al. Effectiveness of Covid-19 vaccines against the B.1.617.2 (Delta) variant. N Engl J Med 2021;385:585–94. 34289274 10.1056/NEJMoa2108891 PMC8314739

[32] Planas D, Veyer D, Baidaliuk A, et al. Reduced sensitivity of SARS-CoV-2 variant Delta to antibody neutralization. Nature 2021;596:276–80. 34237773 10.1038/s41586-021-03777-9

[33] Liu C, Ginn HM, Dejnirattisai W, et al. Reduced neutralization of SARS-CoV-2 B.1.617 by vaccine and convalescent serum. Cell 2021;184:4220–36.e13. 34242578 10.1016/j.cell.2021.06.020 PMC8218332

[34] Mlcochova P, Kemp SA, Dhar MS, The Indian SARS-CoV-2 Genomics Consortium (INSACOG), et al. SARS-CoV-2 B.1.617.2 Delta variant replication and immune evasion. Nature 2021;599:114–9. 34488225 10.1038/s41586-021-03944-y PMC8566220

[35] Gao SJ, Guo H, Luo G. Omicron variant (B.1.1.529) of SARS-CoV-2, a global urgent public health alert! J Med Virol 2022;94:1255–6. 34850421 10.1002/jmv.27491 PMC9015397

[36] Araf Y, Akter F, Tang Y-D, et al. Omicron variant of SARS-CoV-2: Genomics, transmissibility, and responses to current COVID-19 vaccines. J Med Virol 2022;94:1825–32. 35023191 10.1002/jmv.27588 PMC9015557

[37] Liu L, Iketani S, Guo Y, et al. Striking antibody evasion manifested by the Omicron variant of SARS-CoV-2. Nature 2022;602:676–81. 35016198 10.1038/s41586-021-04388-0

[38] Dejnirattisai W, Shaw RH, Supasa P, Com-COV2 study group, et al. Reduced neutralisation of SARS-CoV-2 omicron B.1.1.529 variant by post-immunisation serum. Lancet 2022;399:234–6. 34942101 10.1016/S0140-6736(21)02844-0 PMC8687667

[39] Cao Y, Wang J, Jian F, et al. Omicron escapes the majority of existing SARS-CoV-2 neutralizing antibodies. Nature 2022;602:657–63. 35016194 10.1038/s41586-021-04385-3 PMC8866119

[40] Hoffmann M, Krüger N, Schulz S, et al. The Omicron variant is highly resistant against antibody-mediated neutralization: implications for control of the COVID-19 pandemic. Cell 2022;185:447–56.e11. 35026151 10.1016/j.cell.2021.12.032 PMC8702401

[41] Nemet I, Kliker L, Lustig Y, et al. Third BNT162b2 vaccination neutralization of SARS-CoV-2 omicron infection. N Engl J Med 2022;386:492–4. 34965337 10.1056/NEJMc2119358 PMC8823651

[42] Garcia-Beltran WF, St. Denis KJ, Hoelzemer A, et al. mRNA-based COVID-19 vaccine boosters induce neutralizing immunity against SARS-CoV-2 Omicron variant. Cell 2022;185:457–66.e4. 34995482 10.1016/j.cell.2021.12.033 PMC8733787

[43] Owen DR, Allerton CMN, Anderson AS, et al. An oral SARS-CoV-2 M pro inhibitor clinical candidate for the treatment of COVID-19. Science 2021;374:1586–93. 34726479 10.1126/science.abl4784

[44] Pavan M, Bolcato G, Bassani D, et al. Supervised Molecular Dynamics (SuMD) Insights into the mechanism of action of SARS-CoV-2 main protease inhibitor PF-07321332. J Enzyme Inhib Med Chem 2021;36:1646–50. 34289752 10.1080/14756366.2021.1954919 PMC8300928

[45] Hammond J, Leister-Tebbe H, Gardner A, et al. Oral nirmatrelvir for high-risk, nonhospitalized adults with covid-19. N Engl J Med 2022;386:1397–408 35172054 10.1056/NEJMoa2118542 PMC8908851

[46] Investigation of SARS-CoV-2 variants: technical briefings - GOV.UK. https://www.gov.uk/government/publications/investigation-of-sars-cov-2-variants-technical-briefings

[47] Lacek KA, et al. Identification of a novel SARS-CoV-2 delta-omicron recombinant virus in the United States. bioRxiv 2022.

[48] Aksamentov I, Roemer C, Hodcroft E, Neher R. Nextclade: clade assignment, mutation calling and quality control for viral genomes. J Open Source Softw 2021;6:3773.

[49] Molecular Operating Environment (MOE), 2019.01; Chemical Computing Group ULC, 1010 Sherbooke St. West, Suite #910, Montreal, QC, Canada, H3A 2R7. 2021. https://www.chemcomp.com/Research-Citing_MOE.htm

[50] Zhou D, Duyvesteyn HME, Chen C-P, et al. Structural basis for the neutralization of SARS-CoV-2 by an antibody from a convalescent patient. Nat Struct Mol Biol 2020;27:950–8. 32737466 10.1038/s41594-020-0480-y

[51] Wang Y, Liu C, Zhang C, et al. Structural basis for SARS-CoV-2 Delta variant recognition of ACE2 receptor and broadly neutralizing antibodies. Nat Commun 2022;13:871. 35169135 10.1038/s41467-022-28528-w PMC8847413

[52] Yin W, Xu Y, Xu P, et al. Structures of the Omicron spike trimer with ACE2 and an anti-Omicron antibody. Science 2022;375:1048–53. 35133176 10.1126/science.abn8863 PMC8939775

[53] Case DA, Darden TA, Cheatham TE, et al. Amber 10. San Francisco: University of California; 2008.

[54] Humphrey W, Dalke A, Schulten K. VMD: Visual molecular dynamics. J Mol Graph 1996;14:33–8. 8744570 10.1016/0263-7855(96)00018-5

[55] Huang Y, Yang C, Xu X, et al. Structural and functional properties of SARS-CoV-2 spike protein: potential antivirus drug development for COVID-19. Acta Pharmacol Sin 2020;41:1141–9. 32747721 10.1038/s41401-020-0485-4 PMC7396720

[56] Li M-Y, Li L, Zhang Y, Wang X-S. Expression of the SARS-CoV-2 cell receptor gene ACE2 in a wide variety of human tissues. Infect Dis Poverty 2020;9:45. 32345362 10.1186/s40249-020-00662-x PMC7186534

[57] Jackson CB, Farzan M, Chen B, Choe H. Mechanisms of SARS-CoV-2 entry into cells. Nat Rev Mol Cell Biol 2022;23:3–20. 34611326 10.1038/s41580-021-00418-x PMC8491763

[58] Lan J, Ge J, Yu J, et al. Structure of the SARS-CoV-2 spike receptor-binding domain bound to the ACE2 receptor. Nature 2020;581:215–20. 32225176 10.1038/s41586-020-2180-5

[59] Zhu C, He G, Yin Q, et al. Molecular biology of the SARs-CoV-2 spike protein: A review of current knowledge. J Med Virol 2021;93:5729–41. 34125455 10.1002/jmv.27132 PMC8427004

[60] Turkahia Y, et al. Pandemic-scale phylogenomics reveals elevated recombination rates in the SARS-CoV-2 Spike Region. bioRxiv 2021.

[61] Zhang B-Z, Hu Y-F, Chen L-L, et al. Mining of epitopes on spike protein of SARS-CoV-2 from COVID-19 patients. Cell Res 2020;30:702–4. 32612199 10.1038/s41422-020-0366-x PMC7327194

[62] Hadj Hassine I. Covid‐19 vaccines and variants of concern: a review. Rev Med Virol 2021;e2313. 10.1002/rmv.2313 PMC8646685 34755408

[63] https://covdb.stanford.edu/sierra/sars2/by-patterns/

[64] VanBlargan LA, Errico JM, Halfmann PJ, et al. An infectious SARS-CoV-2 B.1.1.529 Omicron virus escapes neutralization by therapeutic monoclonal antibodies. Nat Med 2022;28:490–5. 35046573 10.1038/s41591-021-01678-y PMC8767531

[65] Meng B, Kemp SA, Papa G, COVID-19 Genomics UK (COG-UK) Consortium, et al. Recurrent emergence of SARS-CoV-2 spike deletion H69/V70 and its role in the Alpha variant B.1.1.7. Cell Rep 2021;35:109292. 34166617 10.1016/j.celrep.2021.109292 PMC8185188

[66] Hou YJ, Chiba S, Halfmann P, et al. SARS-CoV-2 D614G variant exhibits efficient replication ex vivo and transmission in vivo. Science 2020;370:1464–8. 33184236 10.1126/science.abe8499 PMC7775736

[67] Weisblum Y, Schmidt F, Zhang F, et al. Escape from neutralizing antibodies 1 by SARS-CoV-2 spike protein variants. Elife 2020;9:1. 10.7554/eLife.61312 PMC7723407 33112236

[68] Lubinski B, Fernandes MHV, Frazier L, et al. Functional evaluation of the P681H mutation on the proteolytic activation of the SARS-CoV-2 variant B.1.1.7 (Alpha) spike. iScience 2022;25:103589. 34909610 10.1016/j.isci.2021.103589 PMC8662955

[69] Zahradník J, Marciano S, Shemesh M, et al. SARS-CoV-2 variant prediction and antiviral drug design are enabled by RBD in vitro evolution. Nat Microbiol 2021;6:1188–98. 34400835 10.1038/s41564-021-00954-4

[70] Berman HM, Westbrook J, Feng Z, et al. The Protein Data Bank. Nucleic Acids Res 2000;28:235–42. 10592235 10.1093/nar/28.1.235 PMC102472

[71] Masre SF, Jufri NF, Ibrahim FW, Abdul Raub SH. Classical and alternative receptors for SARS-CoV-2 therapeutic strategy. Rev Med Virol 2021;31:1–9. 10.1002/rmv.2207 PMC7883063 33368788

[72] Sartore G, et al. In silico evaluation of the interaction between ACE2 and SARS-CoV-2 Spike protein in a hyperglycemic environment. Sci. Rep 2021;11:22860. 34819560 10.1038/s41598-021-02297-w PMC8613179

[73] Jin Z, Du X, Xu Y, et al. Structure of Mpro from SARS-CoV-2 and discovery of its inhibitors. Nat 2020;582:289–93. 10.1038/s41586-020-2223-y 32272481

[74] Xia B, Kang X. Activation and maturation of SARS-CoV main protease. Protein Cell 2011;2:282–90. 21533772 10.1007/s13238-011-1034-1 PMC4875205

[75] Snijder EJ, Decroly E, Ziebuhr J. The Nonstructural Proteins Directing coronavirus RNA synthesis and processing. Adv Virus Res 2016;96:59–126. 27712628 10.1016/bs.aivir.2016.08.008 PMC7112286

[76] Fornasier E, Macchia ML, Giachin G, et al. A new inactive conformation of SARS-CoV-2 main protease. Acta Crystallogr D Struct Biol 2022;78:363–78. 35234150 10.1107/S2059798322000948 PMC8900819

[77] Wu F, Zhao S, Yu B, et al. A new coronavirus associated with human respiratory disease in China. Nat 2020;579:265–9. 10.1038/s41586-020-2008-3 PMC7094943 32015508

[78] Anand K, Palm GJ, Mesters JR, et al. Structure of coronavirus main proteinase reveals combination of a chymotrypsin fold with an extra alpha-helical domain. Embo J 2002;21:3213–24. 12093723 10.1093/emboj/cdf327 PMC126080

[79] Chen H, Wei P, Huang C, et al. Only one protomer is active in the dimer of SARS 3C-like proteinase. J Biol Chem 2006;281:13894–8. 16565086 10.1074/jbc.M510745200 PMC7990651

[80] Anand K, Ziebuhr J, Wadhwani P, et al. Coronavirus main proteinase (3CLpro) structure: basis for design of anti-SARS drugs. Science 2003;300:1763–7. 12746549 10.1126/science.1085658

[81] Ullrich S, Nitsche C. The SARS-CoV-2 main protease as drug target. Bioorg Med Chem Lett 2020;30:127377. 32738988 10.1016/j.bmcl.2020.127377 PMC7331567

[82] Bassani D, Pavan M, Bolcato G, et al. Re-exploring the ability of common docking programs to correctly reproduce the binding modes of non-covalent inhibitors of SARS-CoV-2 protease MPRO. Pharmaceuticals 2022;15:180. 35215293 10.3390/ph15020180 PMC8878732

[83] Bissaro M, Bolcato G, Pavan M, et al. Inspecting the mechanism of fragment hits binding on SARS-CoV-2 Mpro by using Supervised Molecular Dynamics (SuMD) simulations. Chem Med Chem 2021;16:2075–81. 33797868 10.1002/cmdc.202100156 PMC8250706

[84] Luttens A, Gullberg H, Abdurakhmanov E, et al. Ultralarge virtual screening identifies SARS-CoV-2 main protease inhibitors with broad-spectrum activity against coronaviruses. J Am Chem Soc 2022;144:2905–20. 35142215 10.1021/jacs.1c08402 PMC8848513

[85] Zhang C-H, Stone EA, Deshmukh M, et al. Potent noncovalent inhibitors of the main protease of SARS-CoV-2 from molecular sculpting of the drug perampanel guided by free energy perturbation calculations. ACS Cent Sci 2021;7:467–75. 33786375 10.1021/acscentsci.1c00039 PMC7931627

[86] Flynn JM, et al. Comprehensive fitness landscape of SARS-CoV-2 M^pro^ reveals insights into viral resistance mechanisms. bioRxiv 2022. 10.7554/eLife.77433 PMC9323007 35723575

[87] Greasley SE, et al. Structural basis for Nirmatrelvir in vitro efficacy against SARS-CoV-2 variants. bioRxiv 2022. 10.1016/j.jbc.2022.101972 PMC9023115 35461811

[88] Xue X, Yang H, Shen W, et al. Production of authentic SARS-CoV M(pro) with enhanced activity: application as a novel tag-cleavage endopeptidase for protein overproduction. J Mol Biol 2007;366:965–75. 17189639 10.1016/j.jmb.2006.11.073 PMC7094453

[89] Ho B-L, Cheng S-C, Shi L, et al. Critical assessment of the important residues involved in the dimerization and catalysis of MERS coronavirus main protease. PLoS One 2015;10:e0144865. 26658006 10.1371/journal.pone.0144865 PMC4682845

